# Employing supervised machine learning algorithms for classification and prediction of anemia among youth girls in Ethiopia

**DOI:** 10.1038/s41598-024-60027-4

**Published:** 2024-04-20

**Authors:** Alemu Birara Zemariam, Ali Yimer, Gebremeskel Kibret Abebe, Wubet Tazeb Wondie, Biruk Beletew Abate, Addis Wondmagegn Alamaw, Gizachew Yilak, Tesfaye Masreshaw Melaku, Habtamu Setegn Ngusie

**Affiliations:** 1 Department of Pediatrics and Child Health Nursing, School of Nursing, College of Medicine and Health Science, Woldia University, Po. Box: 400, Woldia, Ethiopia; 2 Department of Public Health, School of Public Health, College of Medicine and Health Science, Woldia University, Woldia, Ethiopia; 3 Department of Emergency and Critical Care Nursing, School of Nursing, College of Medicine and Health Science, Woldia University, Woldia, Ethiopia; 4 Department of Pediatrics and Child Health Nursing, School of Nursing, College of Medicine and Health Science, Ambo University, Ambo, Ethiopia; 5 Department of Nursing, School of Nursing, College of Medicine and Health Science, Woldia University, Woldia, Ethiopia; 6 Department of Health and Nutrition, Save the Children International, Woldia, Ethiopia; 7 Department of Health Informatics, School of Public Health, College of Medicine and Health Science, Woldia University, Woldia, Ethiopia

**Keywords:** Diseases, Medical research

## Abstract

In developing countries, one-quarter of young women have suffered from anemia. However, the available studies in Ethiopia have been usually used the traditional stastical methods. Therefore, this study aimed to employ multiple machine learning algorithms to identify the most effective model for the prediction of anemia among youth girls in Ethiopia. A total of 5642 weighted samples of young girls from the 2016 Ethiopian Demographic and Health Survey dataset were utilized. The data underwent preprocessing, with 80% of the observations used for training the model and 20% for testing. Eight machine learning algorithms were employed to build and compare models. The model performance was assessed using evaluation metrics in Python software. Various data balancing techniques were applied, and the Boruta algorithm was used to select the most relevant features. Besides, association rule mining was conducted using the Apriori algorithm in R software. The random forest classifier with an AUC value of 82% outperformed in predicting anemia among all the tested classifiers. Region, poor wealth index, no formal education, unimproved toilet facility, rural residence, not used contraceptive method, religion, age, no media exposure, occupation, and having more than 5 family size were the top attributes to predict anemia. Association rule mining was identified the top seven best rules that most frequently associated with anemia. The random forest classifier is the best for predicting anemia. Therefore, making it potentially valuable as decision-support tools for the relevant stakeholders and giving emphasis for the identified predictors could be an important intervention to halt anemia among youth girls.

## Introduction

Anemia refers to a medical condition characterized by a deficiency of red blood cells or a decrease in their size, or a reduction in the concentration of hemoglobin below the usual levels. This condition has the potential to hinder or diminish the blood's ability to effectively transport oxygen throughout the body^1^.

It is commonly found throughout all phases of life, though it is more common among teenage girls and young women^2^. The increased susceptibility of this age group is often attributed to their heightened physiological needs for essential nutrients like iron and folic acid, which are necessary for rapid physical growth. Furthermore, this vulnerability is influenced by the potential loss of these micronutrients due to intestinal parasitic infestations, which are particularly widespread in developing nations^3^.

Globally, more than half of young women have suffered from anemia and approximately one-quarter of them were live in developing countries^4,5^. Anemia burden among young women is also common in sub-Saharan African countries which range from 13.7% in Ethiopia to 61.5% in Ghana^4^. In Ethiopia also has a high incidence of anemia in young women, with an average rate of 29% and a range of 24–38%^6^. Anemia poses as a widespread issue in global health, with connections to negative health outcomes, increased sickness and death rates, and substantial burdens on both health and economic aspects^7,8^. Anemia among young women is a significant issue that hinders their ability to maximize their potential. It diminishes their educational accomplishments, decreases their productivity in the workforce, and impacts their cognitive abilities, stunted growth, delayed puberty, and impaired overall physical development. Moreover, it influences their mental well-being, increases the likelihood of encountering complications during childbirth, experience decreased energy levels, difficulty concentrating, decreased physical performance, and raises the chances of delivering an underweight baby^2,9^. Therefore, early detection, proper diagnosis, and appropriate treatment of anemia are crucial to mitigate these health consequences.

Numerous studies indicate that various factors, including educational attainment, marital status, wealth, nutrition, occupation, type of toilet facility, drinking water source, contraceptive usage, proximity to healthcare facilities, and geographical region, are linked to anemia occurrence among young women^10–12^. During the onset of puberty, there is an increase in cases of anemia caused by inadequate nutrition. This is primarily due to the significant physical and physiological changes experienced by adolescents and young women, which impose greater nutritional demands on their bodies. As a result, they become more susceptible to developing anemia due to nutritional deficiencies^13^. The high prevalence of anemia among young girls can be influenced by various socioeconomic, cultural, and dietary factors such as inadequate intake of essential nutrients, limited economic resources and food insecurity, cultural practices and dietary restrictions, and limited access to healthcare services, including nutritional counseling^9,11,14^.

Although anemia is frequently found in young women, the majority of prior research has concentrated on studying anemia within the reproductive age demographic^9,12,15^ and previous studies analyzing the anemia status of young women in Ethiopia using traditional stastical methods^16^. Nevertheless, there is a lack of existing literature that explores the application of machine learning models for predictive purposes in this particular area. Our contention is that leveraging machine learning models to forecast anemia has the potential to generate substantial advantages and augment the body of empirical evidence.

Machine learning methods possess the capacity to surpass traditional statistical approaches by effectively managing extensive and intricate nonlinear data, operating without the need for preexisting assumptions, and capturing intricate connections among predictors^17,18^. Overall, the utilization of machine learning algorithms for classification and prediction offers numerous advantages, including automation, pattern recognition, adaptability, scalability, objectivity, handling non-linearity, feature selection, and generalization. These makes a powerful tool for addressing a wide range of real-world problems and driving data-driven decision-making^19^. Therefore, in this research, we have utilized eight advanced machine learning techniques, such as association rule mining, to forecast the condition of anemia by utilizing demographic health survey information. Therefore, this study aimed to predict anemia and identify its predictors using the current state- of-the-art ML models. The findings will provide evidence for policymakers to plan scientifically sound programs with integrated interventions to prevent anemia and protect the health of the most vulnerable subgroups of youth girls.

## Methods

### Design, data source, setting, and periods

A nationally representative cross-sectional 2016 Ethiopian Demographic and Health Surveys (EDHS) were conducted. Ethiopia is laying between latitude 3° and 14°N and longitude 33° and 48°E in the horn of Africa and structured in nine regional states, namely Tigray, Afar, Amhara, Benishangul-Gumuz, Gambela, Harari, Oromia, Somali and Southern Nations Nationalities and Peoples of Region and two city administrations (Addis Ababa and Dire Dawa)^20^. Ethiopia is the second-most populous country in Africa next to Nigeria with a population of more than 120 million. The EDHS is a part of the international demographics and health survey (DHS) program led by the United States Agency for International Development, in collaboration with other organizations and host countries. Recorded data were accessed at www.measure dhs.com on request with the assistance of ICF International. The survey took place from January 18 to June 27, 2016 with a multi stage stratified sampling technique on 645 enumeration areas covering the entire nation. The survey had included a nationally representative sample of women (aged 15–49 years) with a total sample size of 15,683 women^21^. In this study, we have included a weighted of 5,642 youth women aged 15–24 as our final sample. Out of all the participants, we have analyzed 19 different features.

### Population of the study

All youth girls aged 15–24 years in Ethiopia were the source populations for this study, whereas all youth girls 15–24 years in the selected enumeration areas (EAs) and whose hemoglobin level recorded were the study populations.

### Sampling procedures

The EDHS sample was stratified and selected in 2 stages cluster sampling procedure. At the first stage, a stratified sample of enumeration areas, 645 EAs (202 in urban) were selected with probability proportional to size: in each stratum, a sample of a predetermined number of EAs is selected independently with probability proportional to the EA measure of size. In the selected EAs, a listing procedure is performed such that all households are listed. At the second stage, after a complete household listing is conducted in each of the selected EAs, a fixed number of households is selected by equal probability systematic sampling in the selected EA^21^. The detailed sampling procedure is available in the EDHS reports from the Measure DHS website (www.dhsprogram.com) for each specific survey.

Sample selection for this study, youths without hemoglobin test result (not tested) and respondents above the age of 24 years were excluded, the final analytic sample of youth girls were 5642 considering the weight.

### Study variables and measurements

#### Outcome variable

We used individual women data sets files, 2016 EDHS, to extract the anemia status of youth girls· Anemia is defined as hemoglobin levels less than 12 g/ dl for non-pregnant and 11 g/dl for pregnant youth girls· It was further categorized into mild, moderate, and severe anemia with a hemoglobin range of 10–11·9 g/dl, 7–9·9 g/dl, and less than 7 g/dl, respectively^14,21^. For the current study we classify it as binary 0 for non-anemic and 1 for anemic merging mild, moderate, severe together.

#### Independent variables

Age Group: Current age of the women and re-coded in to two categories with values of “0” for 15–19, “1” for 20–24. Religion: Recoded in four categories with a value of “0” for Muslim, “1” for Orthodox, “2” for protestant, and “3” for other religious groups (combining catholic, traditional and the other religious categories as youngest women in this category are small in number). Wealth Index: The datasets contained wealth index that was created using principal components analysis coded as “poorest”, “poorer”, “Middle”, “Richer”, and “Richest in the EDHS data set·” For this study we recoded it in to three categories as “poor” (includes the poorest and the poorer categories), “middle”, and “rich” (includes the richer and the richest categories). Occupation: Re-coded in two categories with a value of “0” for not working, and “1” for working. Media exposure: A composite variable obtained by combining whether a respondent reads newspaper/ magazine, listen to radio, and watch television with a value of “0” if women were not exposed to at least one of the three media, and “1” if a woman has access/exposure to at least one of the three media. Educational status: this is the minimum educational level a woman achieved and re-coded into three groups with a value of “0” for no education, “1” for primary education, and “2” for secondary and above (combining secondary and higher education categories together). Source of drinking water: By using the DHS guide it was recoded into two categories as “unimproved” and “improved source”^21,22^. Family size: Recoded in to two categories as 1–4, and greater than or equal 5. Body mass index: re-coded in to three categories with values of 0 for underweight 25 kg/m^2^)^23^. The altitude of the cluster categorized as high and low altitude using 2500 m as reference· Type of place of residence: The variable place of residence recorded as rural and urban in the dataset was used without change. Region: The variable region was coded in to 11 categories in the dataset and we retained without change.

#### Data preprocessing and analytic strategies

**Figure 1:**
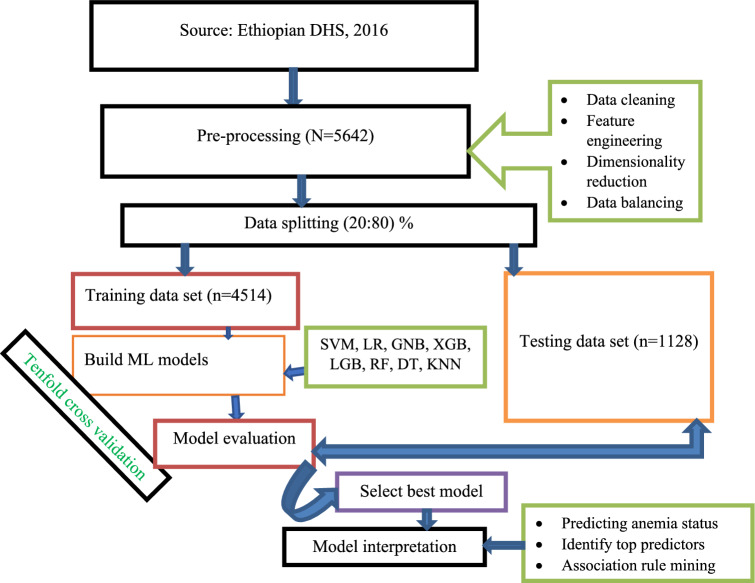
Study workflow diagram.

Preparing raw data for analysis through data pre-processing is essential before building a prediction model in order to improve the model's predictive performance. Data pre-processing involves techniques such as data cleaning, feature engineering, dimensionality reduction, and data splitting^24^. The specific workflow for this study is outlined in Fig. 1.

#### Data cleaning

The initial step in data pre-processing is data cleaning, which involves identifying and removing outliers, handling missing values, and addressing imbalanced categories in the outcome variable. We explored various methods for managing missing data in machine learning, including deletion, imputation, model-based imputation, and domain-specific knowledge. Considering the missingness nature, data amount, assumptions, and the machine learning algorithm used, we have opted to handle missing value in our data set using K-nearest neighbor (KNN) imputation. KNN imputation retains all data, handles outliers, does not assume missingness mechanisms, works for numerical and categorical features, adapts to new data, and minimizes bias while encompassing a wide range of values^25^. In order to identify outliers, we employed various visualization techniques such as scatter plots, box plots, and histograms. These techniques enabled us to detect data points that deviated significantly from the overall pattern. Additionally, we assessed multicollinearity by examining the correlation matrix and considering a correlation value above 0.8 between two pairs of variables as indicative of high correlation.

#### Data balancing

Another data cleaning task was imbalanced data handling. Class imbalance is a significant challenge in data mining and machine learning as it can lead to decreased classification accuracy, particularly for instances belonging to the minority class (45). ML models trained on imbalanced data are typically biased toward the majority class and fail to predict cases that are rare/minority class^26^. To address this issue, researchers have developed various mechanisms. In this study, we employed four balancing methods^27^: under-sampling, over-sampling, adaptive synthetic sampling (ADASYN), and synthetic minority oversampling technique (SMOTE). We aimed to address the imbalance in our dataset and enhance the performance of our predictive model. Initially, we trained our chosen machine learning algorithms using unbalanced data. We then explored various methods such as under-sampling, over-sampling, ADASYN, and SMOTE to balance the data for training the models. Following this, we assessed the performance of the models by comparing accuracy and AUC metrics. In instances where one algorithm showed higher accuracy but lower AUC compared to another, we considered the AUC value for unbalanced data and the accuracy value for balanced data. Accuracy is a suitable metric for balanced classes, while AUC is valuable for imbalanced datasets or when the relative cost of false positives and false negatives is unknown. It is advisable to consider both accuracy and AUC, along with other relevant metrics, to comprehensively understand the model's performance and make informed comparisons between different machine learning algorithms. Taking these factors into account, we selected the balancing technique that demonstrated superior performance for the final prediction.

#### Feature engineering

Feature engineering involves transforming raw data into features that are more suitable for predictive models. In this study, one-hot coding was used to convert categorical variables into numeric values, and label encoding was employed to assign a unique number to each category of variables. Additionally, dimensionality reduction was conducted to decrease the number of input variables for the predictive model, aiming to create a simpler and more effective model for making predictions on new data^28^.

There are two approaches to dimension reduction: feature selection and feature extraction, with the latter being more appropriate for image processing^28^. Feature selection involves choosing the most relevant independent variables that have the greatest impact on predicting the target variable. Feature selection is the appropriate method for our dataset, while feature extraction is typically utilized for datasets involving image processing. There are various well-known methods for feature selection, and it is crucial to carefully consider the predictive performance when selecting a method for ML model. Based on this, we have explored various feature selection methods such as Lasso, PCA; wrapper methods includes forward selection, backward elimination, and recursive feature elimination, correlation-based feature selection, and chi-square test and compared their performance using evaluation metrics^29^. Through this analysis, we have found that Boruta is the most effective feature selection method. We opted for the Boruta-based feature selection method to pinpoint the most important features for our predictive model. Boruta is a wrapper-based technique that uses the random forest classifier algorithm and is known for its unbiased and consistent performance, making it highly effective in selecting key variables^30,31^. Incorporating Boruta with the random forest classifier offers several benefits, including enhanced feature selection, robustness against noise and irrelevant features, reduction of bias in feature importance, and improved interpretability. This combination refines the feature selection process, resulting in better model performance, reduced over fitting, and increased interpretability. However, there are challenges and limitations associated with their use. To address these issues, we have employed various techniques such as L1 or L2 regularization, cross-validation, maintaining an independent test set, parallel processing, analyzing feature importance stability across multiple runs or subsets, recursive feature elimination, balancing false positives and false negatives, and conducting principal component analysis^32^.

Data splitting:- to train the model and validate it on data it has never seen before a simple 80/20 split method in which 80% of samples (4514 respondents data) were used for testing and the rest 20% of respondents (1128 sample) used for testing the model. However, a tenfold cross-validation method was used in this study for model training as it does not waste a lot of data, which is a big advantage when the number of samples is small^33^.

#### Model selection and development

After splitting the data into training and testing sets, we chose appropriate models for training. Since the target variable was categorical, the task involved classification, and we needed to select suitable classifiers for prediction. The dataset falls into the binary classification category, as anemia was divided into two mutually exclusive categories as non-anemic and anemic. To assess the predictive capabilities of ML algorithms in predicting anemia status, we employed eight state-of-the-art algorithms. These algorithms were chosen based on previous research that applied machine learning techniques for classification tasks on EDHS data^17,34–36^. Moreover, the selection of these algorithms were depend on their scalability, interpretability, features number, computational efficiency, data characteristics, type of problem, robustness to noise/outlier, accuracy, bias-variance trade off, and domain expertise. In this study, we utilized the scikit-learn version 1.3.2 packages in Python, implemented within Jupyter Notebook, to employ ML algorithms. The descriptions of eight algorithms are as follows:

(A) Decision tree (DT)

A DT is a non-parametric technique that classifies a data set based on the problem's predictive structure. Decision trees are highly interpretable, efficiently capture nonlinear relationships, handle both categorical and numerical features, relatively robust to outliers and noisy data, handle missing values by utilizing surrogate splits or imputation techniques, and can handle large datasets efficiently^37^. For this study, because of these advantages we have employed DT algorithm to predict the status of anemia among youth girls in Ethiopia. However, DT also have limitations. They can be prone to over fitting, struggle with capturing certain complex relationships that require more sophisticated algorithms, and can be sensitive to small changes in the data, leading to different tree structures.

(B) Random forest (RF)

RF is a type of supervised ML that can be used for classification, regression, and dimension reduction purposes. It is a versatile algorithm used for huge amounts of data and overcoming noise. Random Forest uses an error-minimizing technique to select the variables to split into groups. Random forests are preferred when improved predictive performance, reduced bias, reduction of variance, robustness to noise and outliers, feature importance, and handling high-dimensional data are important considerations for the problem at hand^38,39^. However, RF has some limitations. They can be a black-box model, making it less interpretable or more difficult to interpret compared to individual DT; the ensemble nature of random forests makes it challenging to trace the decision-making process. Additionally, RF may not perform well on datasets with strong linear relationships.

(C) Extreme gradient boost (XG Boost)

XG boost is a DT-based ensemble machine learning algorithm working by a gradient boosting framework. Boosting involves combining weak classifiers to produce a powerful averaged classifier. It can be applied to both classification and prediction problems. XG boost is preferred because of robust to noisy data and outliers, handle high-dimensional datasets, control model complexity and prevent over fitting, handle missing values in the data, saves computational resources, and provides a wide range of hyper parameters^40^. However, XG boost may have higher computational and memory requirements and it also tends to be less interpretable compared to the other algorithms.

(D) Light gradient boosting machine (LGM boost)

Light GBM is a gradient-boosting framework that works by combining multiple learners usually DT to create a strong predictive model and reduce memory usage. Light GBM is generally faster and more memory-efficient, making it suitable for large datasets than XG boost^41^. Light GBM is preferred when efficiency, scalability, handling high-dimensional data, handling categorical features, advanced boosting techniques, regularization techniques, feature importance, handling imbalanced datasets, and flexibility are important considerations for the problem at hand.

(E) Support vector machine (SVM)

SVM is a set of supervised learning methods used for classification, regression, and outlier detection. SVMs are preferred when dealing with high-dimensional spaces, robustness to outliers, nonlinearity, margin maximization, memory efficiency, and small to medium-sized datasets are important considerations for the problem at hand^42^. However, SVMs may have limitations in terms of scalability to large datasets and computational efficiency, especially when using non-linear kernels. Besides, SVMs may not perform well when the dataset is imbalanced, or when the classes are overlapping and not well-separated.

(F) Logistic regression (LR)

LR is a supervised ML algorithm used to solve classification issues. It is a parametric method that assumes a Bernoulli distribution of the target variable and the independence of the observations^42^.

(G) K-nearest Neighbor (KNN)

KNN is a non-parametric, robust, and adaptable supervised ML primarily used for classification problem. This approach keeps track of all existing cases and categorizes new ones using a similarity score with a distance function and the majority vote of its neighbors. KNN is preferred when dealing with nonlinear relationships, interpretability, robustness to outliers, handling imbalanced datasets, no explicit training step, flexibility, and datasets with varying densities are important considerations for the problem at hand^43^. However, KNN has limitations. It can be computationally expensive, especially when dealing with large datasets or high-dimensional feature spaces. Besides, KNN is sensitive to the choice of the distance metric, and the optimal value of K needs to be determined through experimentation or cross-validation.

(H) Gaussian Naïve Bayes (GNB).

NB is a collection of ML algorithms built based on Bayes theorem which has two basic assumptions. The first one is every pair of features should be independent of each other and the second assumption is the feature must have an equal contribution to the outcome prediction. GNB is preferred when efficiency, simplicity, handling continuous features, small training sets, text classification, and the feature independence assumption are important considerations for the problem at hand^44^. However, GNB may not perform well in cases where the two assumptions are severely violated. It may struggle with datasets where the features have strong dependencies or when the decision boundary is complex.

### Model training and evaluation

After dividing the data into training and testing sets, we selected appropriate models for training, focusing on classifiers suitable for the categorical target variable. The dataset involved binary classification for anemia, so we utilized eight machine learning algorithms including logistic regression, random forest, K-nearest neighbor, support vector machine; Gaussian Naïve Bayes, eXtreme gradient boosting, decision tree, and light gradient boost classifiers. These choices were based on previous research using machine learning techniques on EDHS data.

Following model selection, we trained the selected classifiers with both balanced and unbalanced data. The best predictive model was then chosen and trained with balanced training data for the final prediction on unseen test data. To evaluate the performance of the final model, we used a confusion matrix and receiver operating characteristic (ROC) curve with metrics such as accuracy, sensitivity (recall), specificity, F1 score, and area under the curve (AUC). The AUC was considered the main performance metric, providing an overall assessment of the model's performance at different classification thresholds. The confusion matrix allowed us to extract one-dimensional performance metrics such as True Positive (TP), True Negative (TN), False Positive (FP), and False Negative (FN)^26^.

Ultimately, the choice of the best evaluation metrics should be driven by the specific context requirements, trade-off between different evaluation metrics, benchmark and standard on the same field, model interpretability, problem type, data characteristics, and goals of the task at hand. For instance, accuracy is suitable when the distribution of classes is balanced and the costs of misclassifying instances are equal. On the other hand, sensitivity is especially valuable in situations where the classes are imbalanced, meaning there is a high cost associated with missing positive instances, and in applications where it is crucial to detect or mitigate risks early on^45^. Additionally, the ROC curve is beneficial in imbalanced class scenarios for selecting appropriate thresholds and for comparing different models^46^. Therefore, it’s crucial to carefully evaluate and select the metrics that best align with the problem type, data characteristics, and objectives to effectively assess the model's performance^47–49^.

In addition to the standard metrics, tenfold cross-validation techniques were employed to further evaluate the model's performance^50^. Tenfold cross-validation involves dividing the data into ten subsets and training and evaluating the model ten times, each time using a different combination of nine subsets for training and one subset for evaluation^51^. The research also carried out a comprehensive examination of hyper parameters with the aim of enhancing and optimizing the model's performance. Various methods such as grid search, random search, and Bayesian optimization were systematically employed to discover the most effective hyper parameter configurations. The choice of these methods is depend on various factors such as the size of the search space, the available computational resources, and the desired balance between exploration and exploitation. Grid search is a simple and exhaustive method but can be computationally expensive. Random search is less intensive but may require more iteration. Bayesian optimization is efficient and effective for complex search spaces but may require additional setup and computational resources. Suitability of each tuning method also depends on the specific machine learning algorithm being used and the characteristics of the dataset. Experimentation and evaluation of different methods on the validation set is recommended to identify the most effective approach for hyper parameter tuning^52^. Therefore, the authors tried all techniques considering their advantages to select the best tuning technique based on their performance metrics. Additionally, to enhance the precision and reliability of the model used in this study, calibration was conducted. By fine-tuning the model through calibration, its ability to accurately predict the desired outcome was significantly improved.

### Model interpretability

Researchers have highlighted the potential of integrating SHAP (SHapley Additive exPlanations) values and association rule mining to accomplish various goals^53^. When the aim is to uncover concealed patterns and connections within the data, association rule mining proves to be a more suitable method. On the other hand, when the objective is to comprehend how different features influence the model's predictions, SHAP analysis emerges as a more appropriate choice^53,54^. To gain a thorough understanding of the data and analyze the factors that influence the prediction of anemia, we employed a range of techniques. Firstly, we calculated the average SHAP values to assess the overall impact of each feature on the model's predictions. This allowed us to gain insights into the relative importance of different variables. SHAP analysis is a widely used method in machine learning for interpreting model predictions and understanding feature importance. It assigns a numerical value, called a SHAP value, to each feature, indicating its contribution to predictions. By calculating SHAP values, practitioners can gain insights into how features influence predictions. Positive values indicate positive contribution, negative values indicate the opposite, and the magnitude represents the strength of influence. SHAP analysis enhances transparency and interpretability, providing a global view of feature importance and explaining individual predictions^55–57^.

Following that, we utilized a waterfall plot to visually represent the cumulative effects of these variables, highlighting their contributions to the overall prediction^58^.

### Association rule mining

For this research, we employed association rule analysis through the Apriori algorithm in R software to identify particular predictor variables linked to anemia. The purpose of this analysis was to uncover connections between categorical attributes and anemia among young girls in Ethiopia, as machine learning algorithms do not inherently reveal which categories have stronger associations with anemia. By investigating frequently occurring patterns and detecting dependencies among attributes, our objective was to comprehend the relationships between different attributes and the level of confidence they hold in predicting anemia. To achieve this, we utilized If/Then statements to uncover these associations^59^. The If/Then association rule is a pair of attributes (X, Y) expressed as X- > Y, where X is the antecedent and Y is the consequent. This rule signifies that if X happens, then Y would also happen. The relationship between X and Y attributes can be categorized based on the lift value. A lift value of 1 indicates an uncorrelated rule, meaning that X and Y appearing at the same time belong to independent random events and have no special significance. If the lift value is less than 1, it indicates a negative correlation rule, where the occurrence of X reduces the occurrence of Y. On the other hand, if the lift value is greater than 1, it indicates a positive correlation rule, where the occurrence of X promotes the occurrence of Y^60^.

### Ethical considerations and consent to participate

The CSA received the ethical clearance for the 2016 EDHS survey from the Ethiopian Health and Nutrition Research Institute Review Board and the National Research Ethics Review Committee at the Ministry of Science and Technology. Moreover, they confirmed that their research has been performed in accordance with the declaration of Helsinki and the Central Statistical Agency (CSA) obtained written informed consent from the respondents. The authors obtained approval from the DHS Program to access and utilize their data for our study.

## Results

### Socio-demographic characteristics of study participants

A total weighted sample of 5642 youth girls was included in this study. Among this, 1435 (25.43%) of the participants had anemia. More than half (54.34%) of the respondents were aged 15–19 years and nearly to half (49.52%) of respondents had completed primary education. Regarding wealth status and religion, 51.13% of respondents were from rich households and about 40.77% of respondents were orthodox Christian followers. More than two-third (70.6%) of the respondents was from households with an unimproved toilet facility and the majority (64.53%) of the respondent was rural dwellers. Concerning family size, the majority (53.54%) of women were from a family size of greater or equal to 5 and 58.84% of women were not currently working. Regarding media exposure and nutritional status, more than half (53.99%) of respondents were had access to media exposure and two-third of the respondents (66.09%) were had normal nutritional status, respectively (Table 1).

**Table 1:** Socio-demographic characteristics of respondents in Ethiopia, 2016 (N = 5642).

Variables	Categories	Frequency	Percent (%)
Age in years	15–19	3066	54.34
	20–24	2576	45.66
Residence	Urban	2001	35.47
	Rural	3641	64.53
Region	Tigray	668	11.84
	Afar	445	7.89
	Amhara	562	9.96
	Oromia	686	12.16
	Somali	497	8.81
	Benishanguel	383	6.79
	SNNPR	659	11.68
	Gambella	372	6.59
	Harari	307	5.44
	Addis Ababa	682	12.09
	Dire Dawa	381	6.75
Educational status	No formal education	1250	22.16
	Primary	2794	49.52
	Secondary	1174	20.81
	Higher	424	7.52
Religion	Orthodox	2300	40.77
	Muslim	2220	39.35
	Protestant	1042	18.47
	Others	80	1.42
Family size	< 5	2621	46.46
	≥ 5	3021	53.54
Marital status	Unmarried	3419	60.60
	Married	2223	39.40
Wealth index	Poor	2016	35.73
	Medium	727	12.89
	Rich	2899	51.38
Source of drinking water	Protected	4001	70.91
	Unprotected	1641	29.09
Types of toilet facility	Improved	1659	29.40
	Unimproved	3983	70.60
Altitude	Low	3615	64.07
	High	2027	35.93
Occupational status	Not working	3320	58.84
	Working	2322	41.16
Media exposure	Yes	3046	53.99
	No	2596	46.01
Nutritional status	Under nutrition	1587	28.13
	Normal	3726	66.04
	Over nutrition	329	5.83
Contraceptive method	No	4819	85.41
	Yes	823	14.59
Smoking	No	5620	99.61
	Yes	22	0.39
Sex of households	Male	3909	69.28
	Female	1733	30.72

### Machine learning analysis of anemia among youth girls

#### Feature selection

**Figure 2:**
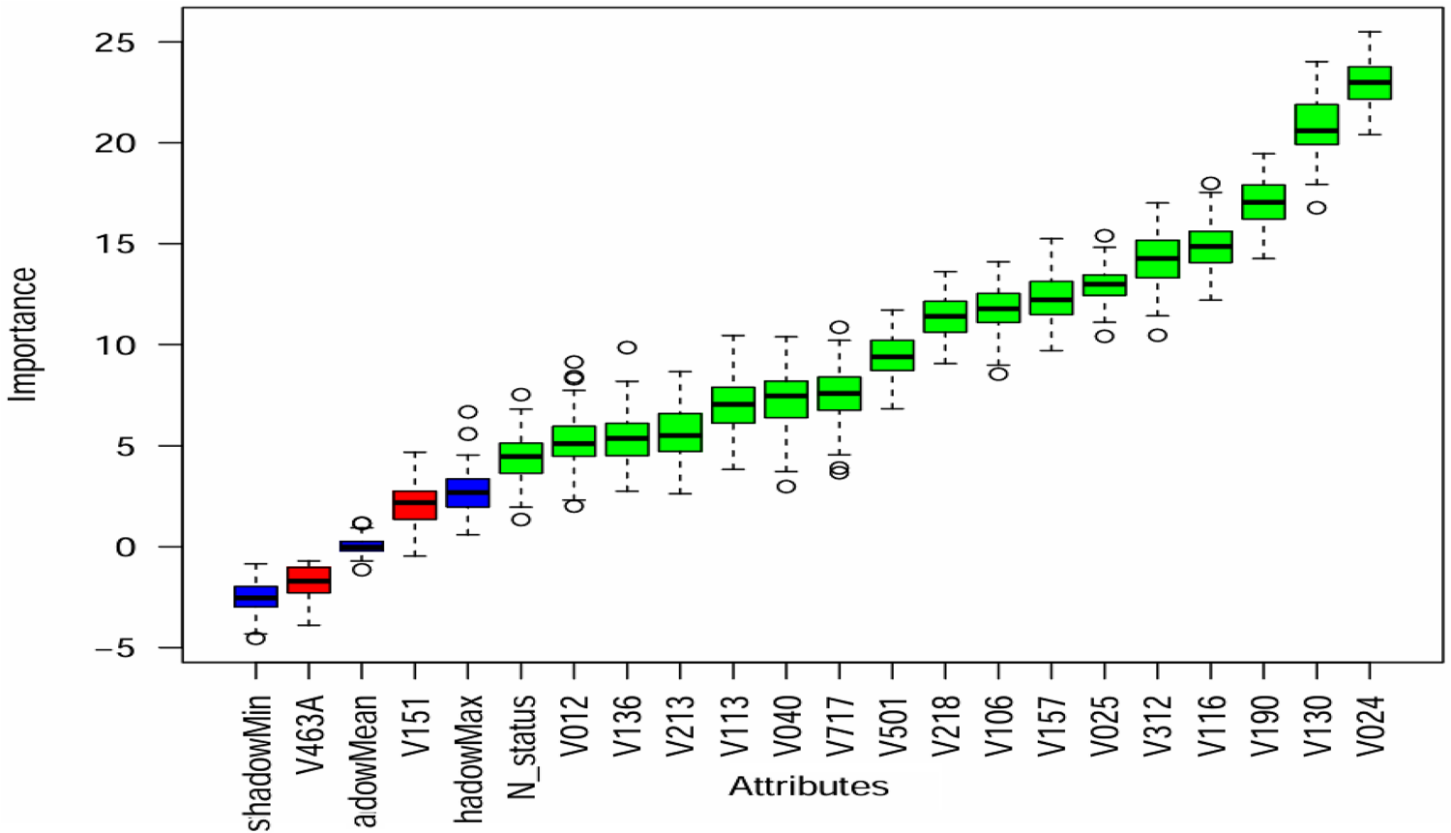
Feature selection using Boruta algorithms. Note: V024-region, V130-religion, V190-wealth index, V116-toilet facility type, V312-contraceptive method, V025-residence, V157-media exposure, V106-educational status, V218-number of children, V501-marrital status, V717-occupation, V040-altitude, V113-source of drinking water, V213-current pregnant, V136-family size, V012-age of respondent, N_status-nutritional status, V151-sex of household, and V463A-smoking.

In Fig. 2, the Boruta algorithm graph is presented, visually representing the significance of variables. Important variables are highlighted in a prominent green color, while unimportant variables are displayed in red.

During our analysis, we excluded household sex and smoking status as they were deemed unimportant by the Boruta algorithm. As a result, we selected the variables identified as important by the Boruta algorithm to predict the anemia status and gain valuable insights into the underlying patterns in the data using association rule mining.

#### Data balancing

We were employed four data balancing techniques such as under-sampling, over-sampling, SMOTE, and ADASYN, and their performance was assessed using an accuracy and AUC value. The balancing techniques that demonstrated high performance were considered for the final prediction. In terms of unbalanced data, the K nearest neighbor achieved an AUC value of 66.5%. Among the under-sampling techniques, oversampling, ADASYN, and SMOTE techniques the random forest model outperformed than the other algorithms with an AUC of 65.7%, 77.4%, 76.8%, and 82.4%, respectively. Considering all the data balancing techniques, SMOTE stood out as the superior method. Table 2 depicted a comparison of different data balancing techniques, including AUC and accuracy value of unbalanced data set.

**Table 2:** Comparison of imbalanced data handling techniques using accuracy and AUC values.

Algorithms	Evaluation metrics	Unbalanced data	Under sampling	Over sampling	SMOTE	ADASYN
SVM	Accuracy	75	62	66	66	64
	AUC	48.3	61.8	50.9	60.8	56.9
GNB	Accuracy	70	62	59	61	60
	AUC	65.7	64.4	60.7	62.7	61
LR	Accuracy	74	64	60	64	61
	AUC	63.6	65.5	62.7	65.4	63.3
DT	Accuracy	66	57	72	68	66
	AUC	51.1	41.1	54.5	49.4	49.4
RF	Accuracy	74	62	76	72	71
	AUC	49	65.7	77.4	82.4	76.8
LGB	Accuracy	76	63	65	66	62
	AUC	55.1	43.4	59.9	71.6	55.8
XGB	Accuracy	72	58	73	71	69
	AUC	54.9	54.6	54.4	58.9	56.7
KNN	Accuracy	75	61	64	64	64
	AUC	66.5	60.5	53	56	54.4

#### Model development and performance evaluation to predict anemia

**Figure 3:**
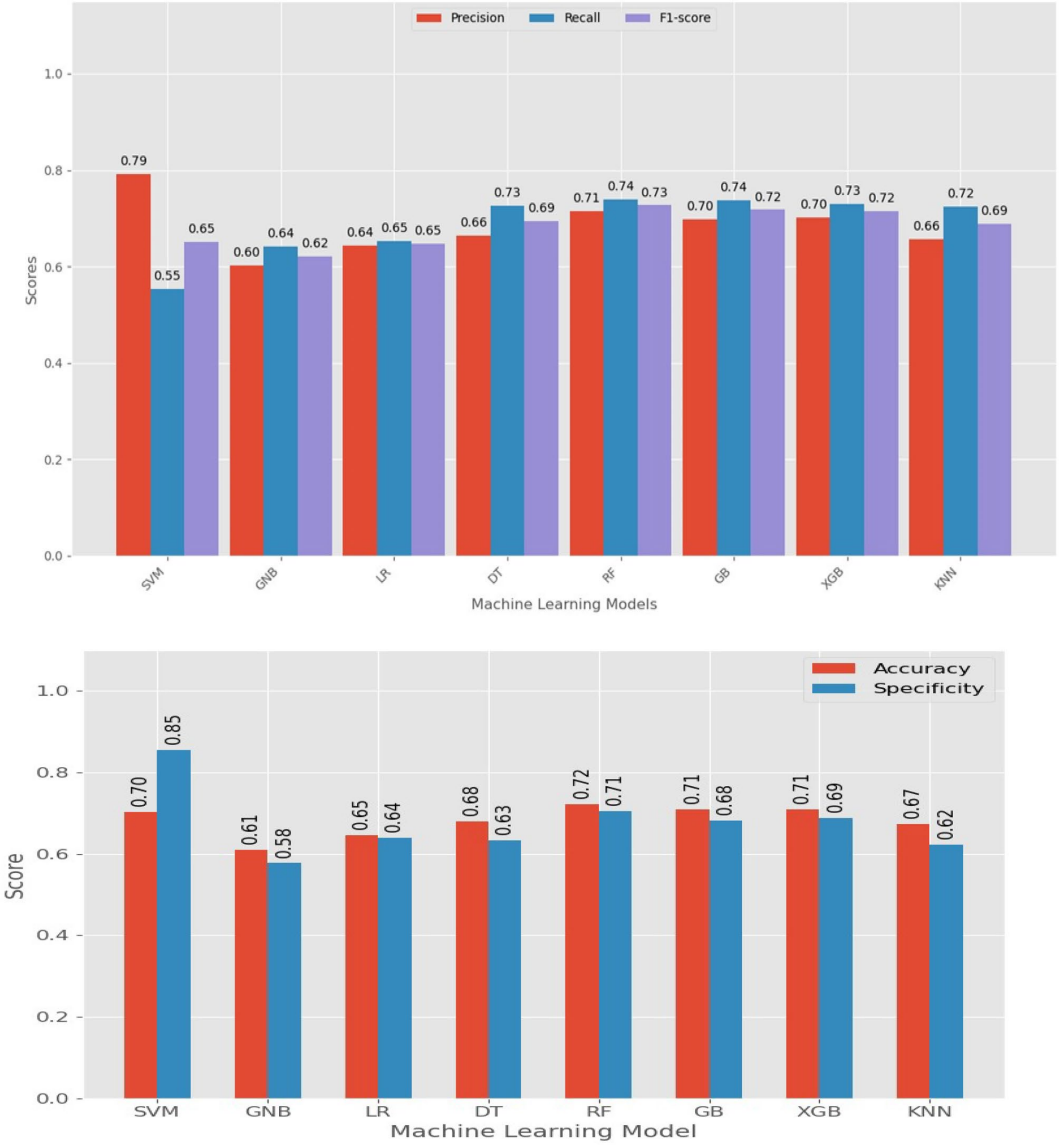
Performance measure of the selected algorithm after data balancing and tuning.

Performance metrics such as accuracy, precision, recall, F1 score, and AUC value were used to evaluate and compare the algorithms' performance. These metrics assessed the overall correctness, ability to correctly predict positive and negative instances, and the algorithm's discriminative power. By utilizing these performance metrics, the researchers conducted a comprehensive evaluation to determine how effectively the algorithms could predict anemia among youth girls in Ethiopia. After comparing the performance metrics of the three tuning techniques we found that the grid search was the best tuning technique achieving highest precision, recall, and f 1 score. Based on the model evaluation metrics results, the top three ML algorithms for classifying anemia status were found to be the random forest classifier, extreme gradient boosting, and support vector machine (Fig. 3).

**Figure 4:**
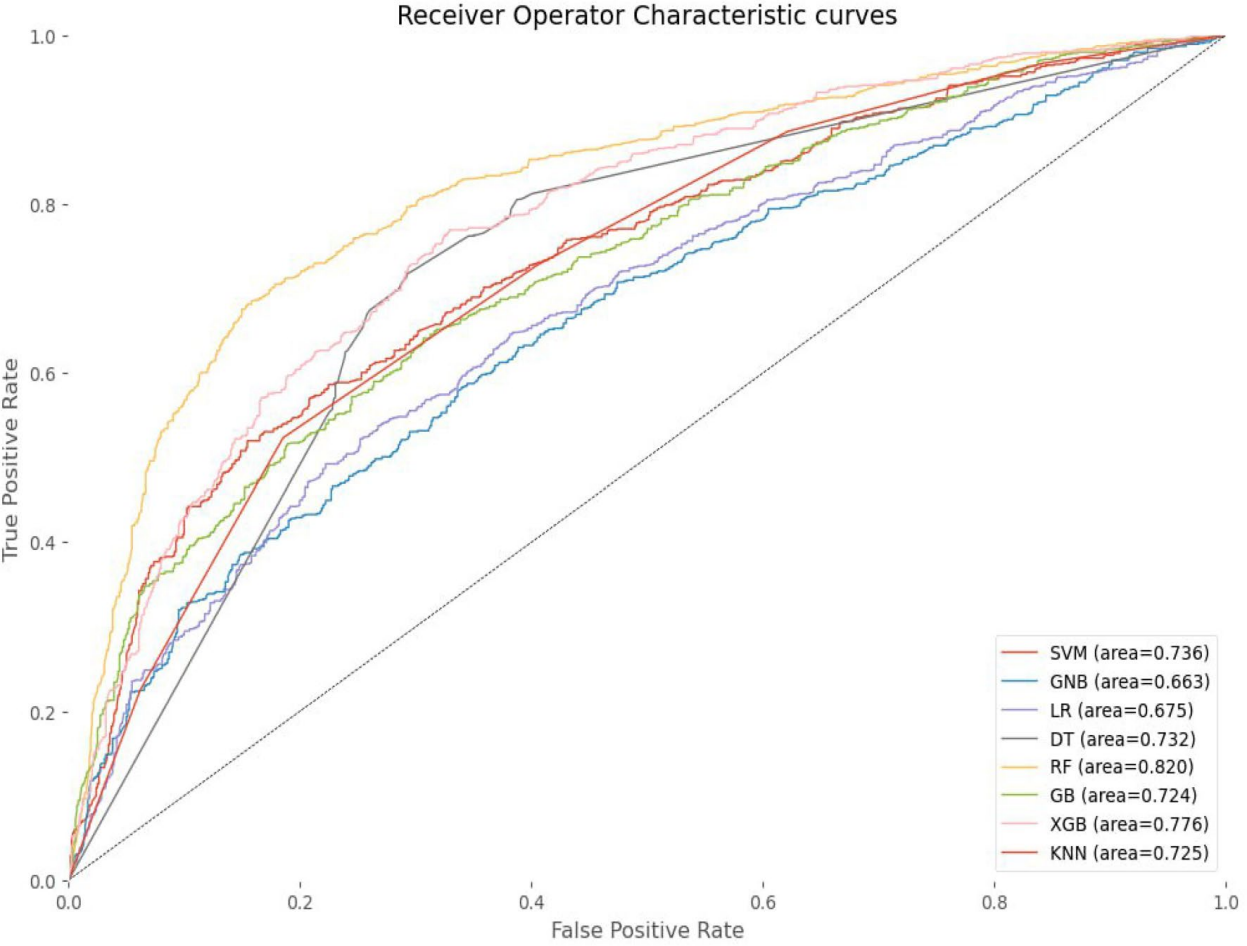
ROC curve analysis of selected machine learning algorithms with balanced data using smote data balancing technique and after optimized hyper parameter tuning.

Figure 4 illustrated the ROC curve analysis of the chosen ML algorithms. The data was balanced using the SMOTE data balancing technique, and further optimization was performed through hyper-parameter tuning. The random forest classifier performed the best among the other tested algorithms, with an AUC of 0.82 followed by the extreme gradient boosting and support vector machine with AUC values of 0.776 and 0.736, respectively, which can be considered an acceptable ROC value.

Likewise, the GNB and LR models obtained AUC values of 0.663 and 0.67 respectively, demonstrating a moderate level of discriminatory capability. Both results indicate that the models are capable of ranking positive instances higher than negative instances approximately 66.3% of the time for GNB and 67% of the time for LR. Furthermore, LR exhibits slightly better discriminatory performance compared to GNB, although the difference is not significant.

### Model interpretability

#### SHAP value interpretation

**Figure 5:**
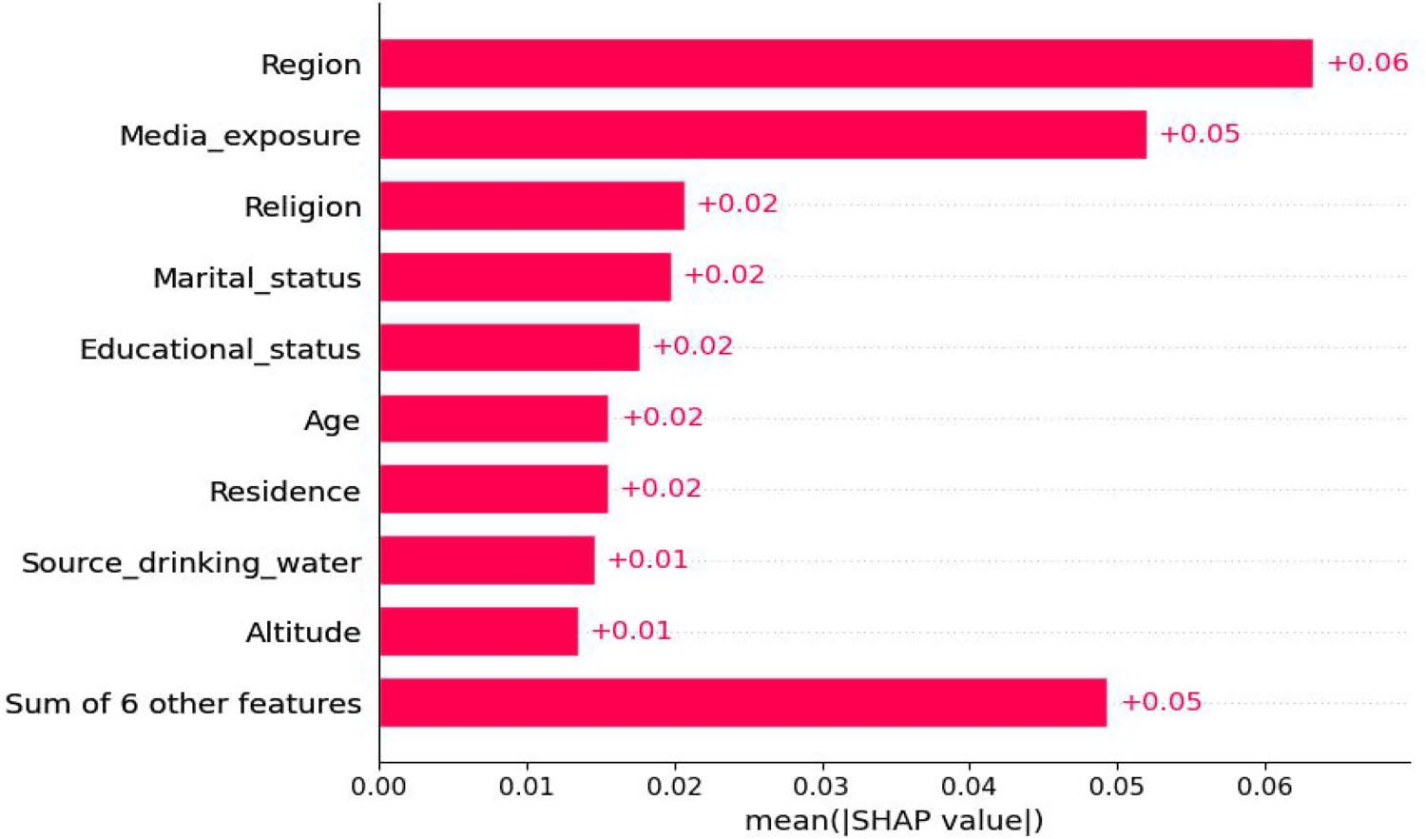
A mean SHAP value report.

Drawing from the results portrayed in Fig. 5, the mean SHAP value report offered valuable insights into the comparative significance of various features in the classification model. Region, media exposure, marital status, educational status, age, religion, and residence were identified as the most influential factors, exerting a substantial impact on the model's predictions. Conversely, the source of drinking water and altitude displayed minimal influence on the classification outcome, as evidenced by their low mean SHAP values. These features contribute less to the model's decision-making process and possess limited importance in predicting the model's outcome.

**Figure 6:**
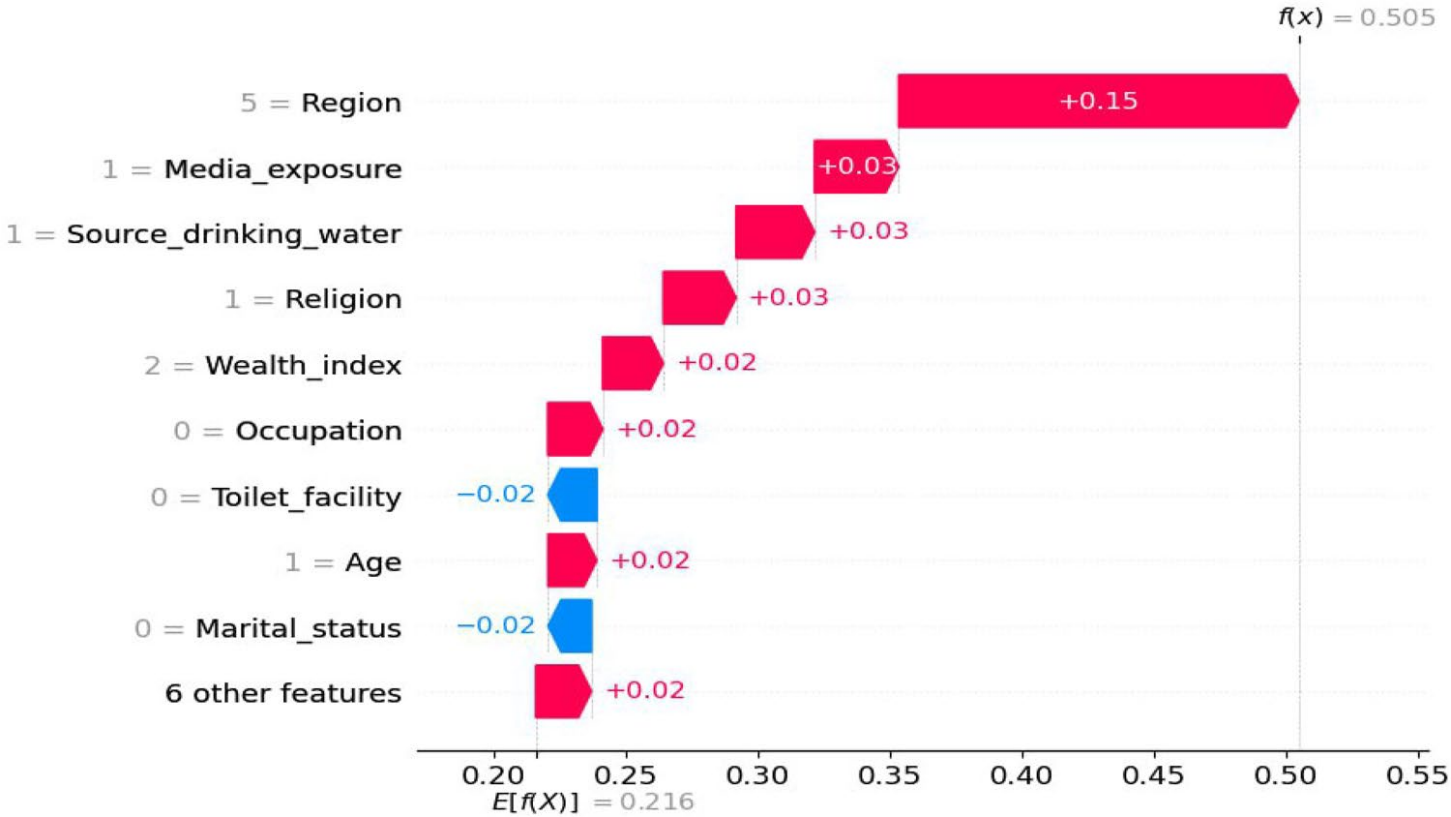
Waterfall plot.

Based on the results presents in Fig. 6, the waterfall plot provided valuable insights into the hierarchy of feature importance when predicting the target variable. The plot highlighted that region had the highest positive impact on the prediction, followed by media exposure, source of drinking water, and religion. Upon further analysis of the waterfall plot, it was observed that age, occupation, and wealth index had a positive contribution to the model's prediction. Higher values of these features tended to increase the predicted outcome, with the exception of type of toilet facility and marital status. These two variables exhibited negative values in the plot, indicating that they had a decreasing effect on the prediction. This suggests that having an improved latrine and being unmarried among young women are associated with a lower predicted outcome in the model, and vice versa.

#### Association rule mining

Using the Apriori algorithm, our research identified the most influential association rules based on their lift values and confidence. These rules provided valuable insights into the probability of anemia among youth girls in Ethiopia. Remarkably, the recurring presence of factors such as age, region, source of water, wealth index, source of water, and educational status in these association rules indicated their consistent association with the likelihood of anemia. These factors exerted a substantial influence on the probability of anemia and warrant attention in efforts aimed at improving maternal health in the region. A total of 46 rules were generated and the top seven association rules were selected and presented as follows:

1. If the youth girl is in the age group of 15–19 years, from Dire Dawa, has a primary education, and has poor wealth index, the probability of being anemic is 96.3% (lift = 3.93).

2. If youth girl from Somali region, has a primary education, has unprotected source of water, has poor wealth status, the probability of being anemic is 92.4% (lift = 3.67).

3. If youth girl from Oromia region, live in high land area, has unprotected source of water, and follows the orthodox religion, the probability of being anemic is 90.0% (lift = 3.54).

4. If youth girl from Oromia region, live in high land area, belongs to a poor wealth index, and follows the orthodox religion, the probability of being anemic is 90.0% (lift = 3.54).

5. If the youth girl is in the age group of 15–19 years, live in Diredawa, has no formal education, and has unimproved toilet facility, the probability of being anemic is 88.9% (lift = 3.21).

6. If the youth girl is live in Somali region, has unprotected source of water, has unimproved toilet facility, and belongs to the poor wealth index, the probability of being anemic is 88.1% (lift = 3.17).

7. If youth girl is rural dwellers, has no formal education, has five and greater than family members, and belongs to the poor wealth index, the likelihood of being anemic is 85.5% (lift = 2.87).

## Discussion

The findings of this research demonstrated the potential of machine learning algorithms in predicting the presence of anemia among youth girls in Ethiopia. This opens up opportunities for the development of automated screening tools and decision support systems that can assist healthcare providers in diagnosing and managing anemia. We have utilized eight different machine learning algorithms, namely Random Forest, Decision Tree, Naive Bayes, KNN, LGB, XGB, SVM, and Logistic Regression, to assess their predictive capabilities. Evaluating their performance, we discovered that all eight algorithms employed in this study achieved ROC values above the optimal threshold. Notably, the random forest classifier, extreme gradient boosting, and support vector machine classifier emerged as the most effective models for classifying anemia status. To maximize the predictive accuracy of the final model, data balancing techniques were employed. After evaluating the performance metrics of the balanced data, the Random Forest model exhibited the best overall performance. The use of a random forest classifier for studying anemia has implications by providing accurate predictive models, insights into risk factors and mechanisms, identification of vulnerable subgroups, and the potential for integrating machine learning into healthcare systems. These implications pave the way for targeted interventions, personalized healthcare approaches, and improved health outcomes for individuals affected by anemia. This finding aligns with similar studies conducted in Rwanda^18^ and Ethiopia^61^, which revealed that the RF model outperformed the other ML models with a slight difference on the value of evaluation metrics. This slight difference might be due to the size of the data set used for model building across the studies.

The results obtained from analyzing the mean SHAP value report and waterfall plot provided valuable insights regarding the importance of different factors in a classification model used to predict anemia status in young girls. Factors such as region, media exposure, marital status, educational status, age, religion, and residence were found to have a significant impact on the model's predictions and emerged as the most influential features. On the other hand, the source of drinking water and altitude had minimal influence on the classification outcome, as indicated by their low mean SHAP values. These particular features contribute less to the model's decision-making process and hold limited importance in predicting anemia status. Understanding the significance of various features and their influence on the model's predictions can serve as a valuable guide for targeted interventions and policy decisions, ultimately leading to improvements in the health and well-being of young girls in Ethiopia. These insights not only validate existing domain knowledge but also evaluate the effectiveness of the model, resulting in more accurate and impactful interventions related to youth women health in the region.

Regarding this study, the Random Forest model identified several significant predictors for anemia among youth girls. The top twelve important predictors included age, marital status, , type of toilet facility, media exposure, mother's educational status, mother's occupational status, residence, mother's wealth index, region, altitude, family size, and source of drinking water. These factors were found to play a crucial role in predicting anemia among youth girls in the study.

Another aim of this study was to identify the top predictors of anemia among youth girls. To accomplish this, the author utilized the Boruta algorithm to select important features. Out of a total of 17 features included based on the literature; the study identified 15 predictors as important feature to predict anemia. The Boruta algorithm revealed that region, religion, age, marital status, family size, type of toilet facility, media exposure, educational status, occupational status, residence, wealth index, contraceptive use, source of drinking water, and altitude significantly influence the level of anemia among youth girls in Ethiopia. This suggests that ML models may uncover new variables or insights not captured by conventional regression models, which could be valuable for policy decision-making.

The third objective of the study was to use association rule mining with the a priori algorithm to identify patterns and associations between independent predictors and the outcome variable. The top seven rules generated by the best model revealed that being 15–19 years old, having a poor wealth index, having unprotected source of water, using an unimproved toilet facility, living in a rural area, residing in certain regions (Oromia, Somali, Dire Dawa), and having no media exposure were most frequently associated with a high probability of anemia.

The research findings indicated a notable correlation between anemia and media exposure among youth girls. It was observed that individuals who lacked media exposure had a higher probability of being affected by anemia compared to their counterparts. This discovery aligns with a similar investigation conducted in India, underscoring the significance of media exposure as a potential factor influencing the prevalence of anemia among youth girls^62^. The lack of access to information via media and other channels can lead to a deprivation of essential health-related information, such as details about health insurance, disease prevention, and other pertinent health messages. This underscores the importance of media access in ensuring individuals receive vital health information for their well-being^63–65^. This finding aligns with several studies conducted in Ghana, which indicate that children without health insurance face a higher risk of developing anemia compared to those with coverage. Health insurance plays a critical role in facilitating access to healthcare services, including the prevention and treatment of anemia. Uninsured households are more susceptible to financial burdens associated with healthcare costs, which can result in delayed treatment and the exacerbation of health issues, including anemia.

In this study, it was found that marital status was a significant predictor for the occurrence of anemia among youth girls. This finding aligns with a previous research conducted in Ethiopia, which emphasized the influence of marital status on the likelihood of anemia in this population^66^. According to a study conducted in Ethiopia, being married was associated with a higher occurrence of anemia among youth girls^16^. This might be due to early marriage exposes adolescents to the risks associated with pregnancy and childbirth, such as bleeding, which can increase the chances of developing anemia. This can contribute to a higher prevalence of anemia among young girls who marry at an early age^67^. Early marriage and early childbearing can indirectly impact the nutritional status of adolescents, often leading to increased responsibilities and limited educational opportunities for young girls, which can result in inadequate access to proper nutrition. As a consequence, the nutritional status of adolescents may be compromised, potentially leading to a higher prevalence of anemia and other health problems.

Remarkably, the study uncovered a significant correlation between anemia and altitude. The results indicate that young girls residing at higher altitudes have a higher likelihood of developing anemia compared to their counterparts at lower altitudes. This emphasizes the impact of altitude as a contributing factor to the prevalence of anemia among young girls. This finding is supported by a study conducted in Boston, which further strengthens the association between altitude and anemia among this population^68^. As altitude increase, air pressure and oxygen concentration in the atmosphere decrease. Since oxygen is crucial for red blood cell production and hemoglobin synthesis, the reduced oxygen availability at higher altitudes can result in a decline in red blood cell production, ultimately leading to anemia. It is plausible to propose that anemia may be more prevalent at highland altitudes due to the challenging geographic conditions, which can result in food insecurity, leading to iron deficiency anemia^69^.

The results of the study suggest that the wealth status of households is a significant determinant of anemia among young girls. There is a higher prevalence of anemia among youth girls from poor households compared to their counterparts. This finding is consistent with studies conducted in different regions of Ethiopia, including the Oromia regional State^70^ and Southern Ethiopia^71^. These findings can be attributed to various factors, such as food scarcity, poor hygiene and sanitation practices, and inadequate nutrition, which contribute to malnutrition, including iron deficiency anemia.

The study indicates a significant association between anemia and the source of drinking water among young girls. Those who relied on unprotected drinking water were found to have a higher vulnerability to developing anemia compared to those with access to improved drinking water sources. This finding is consistent with studies conducted in Ethiopia^72^, Washington^73^ and India^74^. This is due to the fact that youth girls who lack access to safe drinking water are at a heightened risk of contracting diarrhea, which can contribute to the occurrence of anemia among them. Diarrhea can weaken their immune system, making them more susceptible to various health issues and nutritional deficiencies, including iron deficiency anemia. The presence of diarrhea can compromise their overall health and increase the risk of developing anemia due to associated nutritional deficiencies.

Moreover, this study underscores a significant correlation between the type of toilet facility and anemia among young girls. The utilization of unimproved toilet facilities, which can lead to inadequate stool disposal, raises the likelihood of developing anemia compared to those with access to improved facilities. This finding is consistent with a study conducted in Ethiopia^72^, highlighting the importance of proper sanitation practices in mitigating the risk of anemia among young girls. This association can be attributed to the exposure of young girls to helminths resulting from improperly disposed stool. Such exposure increases the risk of transmitting helminthic diseases, including hookworm infection. Hookworm infection can cause reduced food absorption, decreased appetite, gastrointestinal bleeding, and various complications, ultimately contributing to the development of anemia among young girls.

The study discovered a noteworthy relationship between anemia and the number of family members in households. Young girls living in larger households have a heightened risk of developing anemia compared to those in smaller households. This finding is consistent with studies conducted in Ethiopia^72^, which have reported similar results. The increased risk of anemia in larger households may be attributed to competition for food resources and a higher susceptibility to communicable diseases. These factors can contribute to nutritional deficiencies, particularly iron deficiency anemia, among young girls living in households with a larger number of family members.

The level of education has been identified as a significant predictor of anemia among young girls. Girls who have not received any formal education are more susceptible to developing anemia compared to their counterparts. This emphasizes the importance of education in increasing awareness about nutrition, health, and preventive measures, which can help reduce the risk of anemia among young girls^62^. Studies have consistently shown that girls with no education are associated with a higher risk of anemia, which aligns with a study conducted in West Shewa, Ethiopia^75^. This might be education empowers mothers to effectively manage their environment, including healthcare facilities, collaborate with healthcare professionals, adhere to treatment recommendations, and maintain a clean and healthy environment. Additionally, women with higher levels of education possess greater influence over the health choices made for their young girls.

Similarly, the place of residence is significantly associated with anemia among young girls, as indicated by the findings of this study. Girls living in rural areas face a higher risk of developing anemia compared to their counterparts in urban areas. This finding aligns with a systematic review and meta-analysis conducted in Ethiopia^76^. This finding is also supported by another study conducted in Ethiopia^16^. The increased prevalence of anemia in rural areas can be attributed to factors such as low economic status, limited access to iron-rich foods, lack of information about a balanced diet, and a higher proportion of illiterate individuals. These factors collectively contribute to the occurrence of anemia among young girls residing in rural areas.

Understanding the results of ordinal machine learning (ML) models can be challenging compared to classical regression models because they lack regression coefficients and a clear indication of the impact direction. However, in this study, the researchers used an advanced ML technique called SHAP (Shapley Additive explanations) value analysis to overcome these limitations. SHAP value analysis provides its own logit coefficients, which helps in interpreting the results. ML models often categorize or predict specific factors based on their significance in influencing anemia levels among young girls. To gain a better understanding of the direction of these important factors, it is useful to consult existing empirical literature that utilizes conventional approaches.

ML methods are highly valuable in predicting determinants of population health and other phenomena, which in turn contributes to enhancing policy decisions. They provide insights into the factors that contribute to anemia and assist in making informed decisions to address and prevent anemia among young girls^77,78^. The practical significance of this study lies in its ability to aid in early detection, provide targeted prevention strategies, and guide personalized interventions, and influence resource allocation and policymaking. These implications have the potential to greatly enhance the health outcomes of youth girls in Ethiopia by effectively addressing anemia and reducing its impact on individuals, families, and the healthcare system. As a result, this study introduces new perspectives to the field of anemia intervention among youth girls through its innovative approach, identification of key risk factors, development of accurate prediction models, and proposal of personalized interventions. These contributions provide valuable information for policymakers and program planners and offer insightful guidance for designing focused interventions to improve the health outcomes of youth girls in Ethiopia.

## Strength and limitations of the study

The study incorporates eight supervised ML classification algorithms, providing a comprehensive and robust analysis of the predictive capabilities of different algorithms in order to reveals hidden patterns and relationships in the data that may not be easily identifiable through traditional statistical methods. This deepens the understanding of the factors influencing anemia among youth girls in Ethiopia.

On the other hand, the absence of regression coefficients for each predictor makes it challenging to quantify the strength of their association with anemia which might hinders the ability to precisely measure the impact of individual predictors on the outcome. Certain important factors were not included in the analysis due to the use of secondary data from the DHS. The exclusion of these variables may restrict the comprehensive understanding of the predictors of anemia. The challenges of applying continuous-data methods or machine learning algorithms to discrete variables are also another limitation of our study. Therefore, adapting machine learning algorithms and developing new methods to handle discrete variables effectively is an active area of research in the field.

Besides, cross-validation estimates often exhibit high variability, rendering the statistics uninformative and potentially misleading. Consequently, we suggest that future researchers adopt Bayesian approaches that incorporate prior knowledge and uncertainty estimates to obtain more informative and stable estimates. Additionally, employing an ensemble model by training multiple models on distinct cross-validation splits and combining their predictions is recommended. This approach can help mitigate the issues associated with high variance and enhance the reliability of the results.

Moreover, the findings may not be applicable to other populations or age groups, as our focus was specifically on youth girls in Ethiopia. Future research should explore anemia classification and prediction in diverse demographic groups. Besides, biases or limitations could arise from the feature selection method, only DHS data set used, and limited algorithms included. Therefore, it would be valuable for future research to explore the classification and prediction of anemia using many more algorithms, applied different feature selection methods, and utilize multiple data sources to address these limitations and to investigate additional areas that can enhance our understanding of anemia in this population, ultimately guiding more effective interventions and policies.

## Conclusion and implication of the study

The study highlights the potential of machine learning in accurately predicting the status of anemia among youth girls in Ethiopia. All eight machine learning algorithms performed above the optimal ROC value, indicating their effectiveness. Specifically, the random forest classifier, extreme gradient boosting, and support vector machine demonstrated the highest efficacy in classifying anemia status, with the random forest classifier outperforming the others. These findings carry significant implications for public health interventions in Ethiopia, as ML algorithms can be utilized to develop targeted strategies that promote the adoption of anemia among youth girls.

Through feature importance analysis, several determinant risk factors were identified, including age, marital status, family size, type of toilet facility, media exposure, mother's educational status, mother's occupational status, residence, mother's wealth index, source of drinking water, region, and altitude. Moreover, advanced ML techniques, such as SHAP value logit coefficients, were employed to address the limitations of ordinal ML approaches. The developed ML model, particularly the random forest algorithm, plays a crucial role in informing policy and intervention strategies for the prevention and control of anemia among youth girls.

Our study can have a significant impact on addressing anemia in developing countries. It can enable early detection and diagnosis by analyzing anemia-related data, facilitate remote monitoring and telemedicine to overcome healthcare access limitations, optimize treatment strategies based on patient data, aid in public health planning and resource allocation, recommend personalized interventions, and support data-driven research and policy development^79^. However, successful implementation requires addressing challenges such as data availability, healthcare infrastructure, ethical considerations, and model biases^80^. With proper attention to these challenges, the current study can improve anemia management and outcomes in developing countries.

Policymakers and healthcare providers can use these identified potential factors as indicators to create interventions that meet the specific needs of different subgroups in the population. This tailored approach can enhance the health of youth women and reduce the effects of anemia in areas with limited resources. To put these findings into practice, more research is needed.

## Data Availability

This study dataset used for the current study was publicly available in the DHS repository (https://dhsprogram.com).

## Abbreviations

ARM: Association rule mining
AUC: Area under the curve
CSA: Central stastically agency
DHS: Demographic, and health survey
ML: Machine learning
EDHS: Ethiopian demographic, and health survey
ROC: Receiver operating characteristic curve
SMOTE: Synthetic minority oversampling technique
WHO: World health organization
